# StrucPTM: a database of structurally validated protein modifications and their conformational variation

**DOI:** 10.1093/bioinformatics/btag190

**Published:** 2026-04-22

**Authors:** Seong-gwang Jeon, Jejoong Yoo, Keehyoung Joo, Eunok Paek

**Affiliations:** Department of Computer Science, Hanyang University, Seoul, 04763, Korea; School of Computational Sciences, Korea Institute for Advanced Study, Seoul, 02455, Korea; Center for Advanced Computation, Korea Institute for Advanced Study, Seoul, 02455, Korea; Department of Computer Science, Hanyang University, Seoul, 04763, Korea

## Abstract

**Motivation:**

Post-translational modifications (PTMs) alter functional states and interaction specificity largely through the conformational changes they impose on protein structure. However, most existing resources remain sequence-centric and cannot reveal how chemical modifications reshape 3D structures. To address this gap, we propose a structural database that systematically extracts and contextualizes modification sites within experimentally determined protein structures, providing a foundation for future studies of protein structure, function, and regulatory mechanisms.

**Results:**

We present StrucPTM, a database that extracts modified residues directly from the Protein Data Bank (PDB) structures using atom-level composition rules, substantially expanding coverage beyond annotation-dependent methods. Each validated PTM modification is mapped onto a UniProt entry. The database further characterizes residues using key structural descriptors—including secondary structure, relative solvent accessibility (RSA), and whether the PTM site lies at an interchain interface. All chains associated with the same UniProt ID are compared and grouped into homolog sets based on sequence identity. This emphasizes structural conservation among homologs, allowing PTM-induced conformational deviations to be distinguished from unrelated sequence divergence.

**Availability and implementation:**

StrucPTM offers searchable access, interactive 3D visualization, and homolog-based structural comparison through its web interface: https://prix.hanyang.ac.kr/strucptm. The source code and datasets are permanently archived on Zenodo (DOI: 10.5281/zenodo.18939125) and are accessible via GitHub (https://github.com/HanyangBISLab/StrucPTM.git).

## 1 Introduction

Post-translational modifications (PTMs) influence protein activity, stability, and interaction specificity across diverse cellular processes by altering local chemistry and modulating dynamic behavior (Xin and Radivojac 2012, Bludau et al. 2022). Many of these functional effects ultimately arise from PTM-induced perturbations in the 3D structure, which makes structural characterization critical for understanding their mechanistic functions.

To elucidate the mechanistic roles of PTMs, several large-scale PTM databases have been developed, most of which catalog modification sites at the sequence level (Hornbeck et al. 2019, Zhang et al. 2022, Chung et al. 2025). However, their sequence-centric nature cannot capture how modifications alter folds, interfaces, or conformational ensembles.

Although the Protein Data Bank (PDB) provides the necessary structural information, PTM extraction within PDB entries has relied on heterogeneous and often incomplete annotations, leaving many modified residues unrecognized (Craveur et al. 2014). This limitation makes the structural context difficult to analyze systematically.

To understand the mechanistic impact of PTMs, structural characterization must consider not only where a modification occurs, but also how it alters the surrounding environment. Local structural features such as secondary structure, solvent exposure, and proximity to interchain interfaces provide important context (Kabsch and Sander 1983, Jones and Thornton 1996, Tien et al. 2013). However, these descriptors alone cannot determine whether a PTM influences the global conformation of a protein. Resolving this requires a broader comparison among homologous chains to isolate PTM-specific effects (Xin and Radivojac 2012).

**Figure 1:**
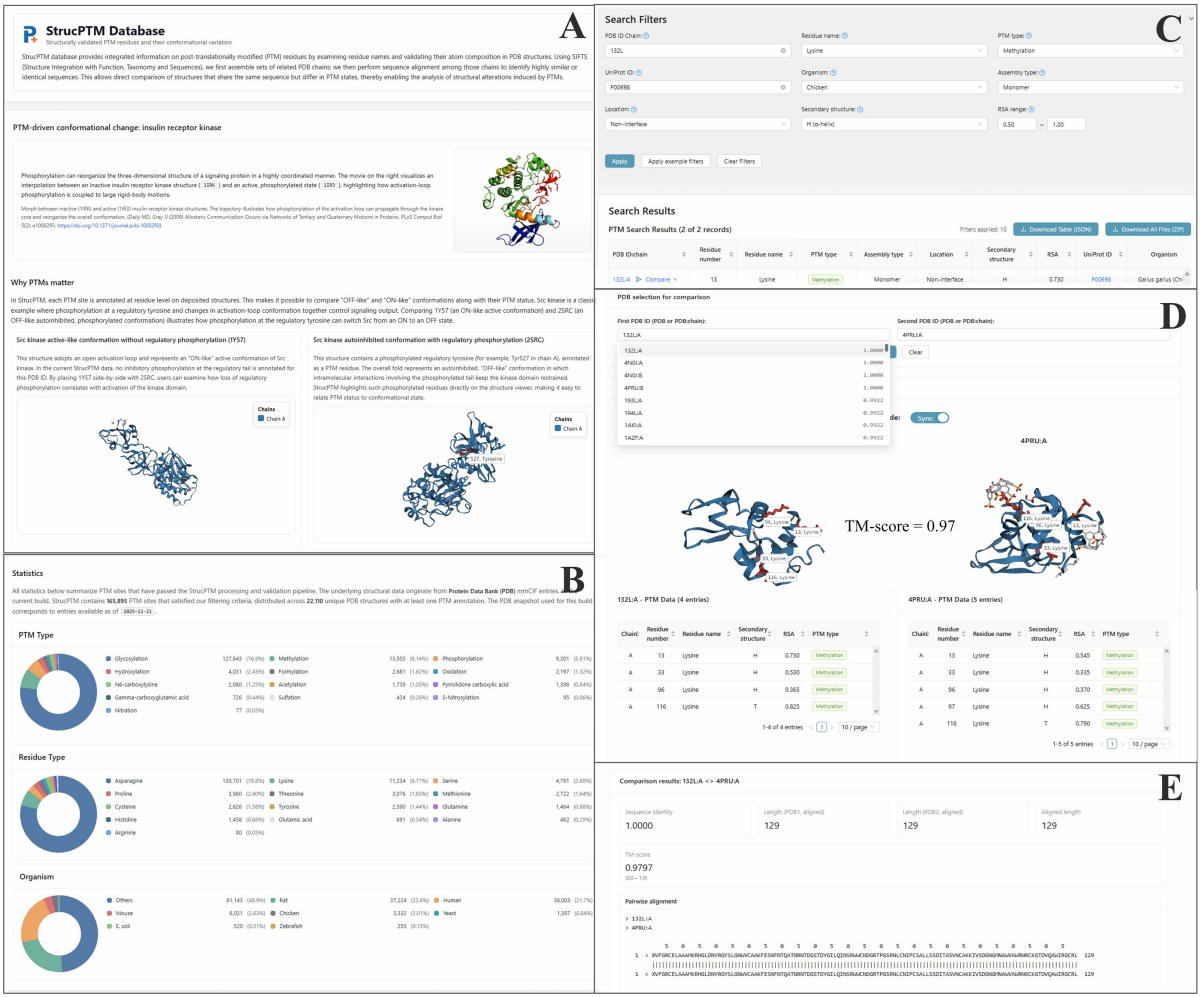
Overview of the StrucPTM web interface. (A) Landing page introducing the StrucPTM database. (B) Statistics summarizing the content of structurally validated PTM entries. (C) Search interface supporting multiparameter filtering. (D) Interactive structure comparison module. (E) Structural comparison results showing TM-score and sequence identity.

These challenges underscore the need for a structural resource that can reliably extract PTMs while providing the contextual information necessary to interpret conformational effects. In response, we introduce StrucPTM, a database that extracts PTMs directly from experimentally determined structures and provides structural context for analyzing PTM-induced conformational variation. Figure 1 provides an overview of the StrucPTM interface, while Fig. S1, available as supplementary data at *Bioinformatics* online, summarizes the overall database construction workflow.

## 2 Results

### 2.1 Database construction

All available mmCIF files in the PDB (Burley et al. 2017) were parsed using Biopython (Cock et al. 2009). Extracting PTMs directly from structural data is challenging because modification fields are heterogeneous and often underspecified.

Previous annotation-based efforts such as PTM-SD (Craveur et al. 2014) captured only explicitly documented PTMs, leaving many chemically verifiable PTMs undiscovered. Therefore, StrucPTM extracts PTMs using structural evidence. An overview of the complete workflow is shown in Fig. S1, available as supplementary data at *Bioinformatics* online. The atom-level composition rules used for validation are summarized in Table S1, available as supplementary data at Bioinformatics online, and a comparison between the counts derived by PTM-SD and counts validated by StrucPTM is provided in Table S2, available as supplementary data at *Bioinformatics* online.

StrucPTM first identifies PTMs by comparing the observed atom sets of nonstandard residues against expected chemical signatures. Glycosylation is uniquely handled by detecting covalent linkages between protein residues and carbohydrate ligands. Chains are mapped to UniProt via SIFTS (Dana et al. 2018) and annotated with secondary structure, RSA, and interface proximity (Table S3, available as supplementary data at *Bioinformatics* online). To ensure robust structural comparisons, sequence identity is calculated over global alignments against full-length sequences. For conformational comparison, chains sharing the same UniProt ID are grouped using a sequence identity threshold of ≥0.75 (Correa Marrero et al. 2025). Finally, all parsed information is integrated into a unified MySQL database.

Across all PTM types, StrucPTM validates 165 895 modification sites, whereas the original PTM-SD procedure reports 36 602 sites (Table S2, available as supplementary data at *Bioinformatics* online). Thus, structure-based identification increases the total number of PTM residues by ∼4.5-fold. The gain is especially pronounced for glycosylation, where StrucPTM recovers 127 643 sites compared to 20 476 in PTM-SD. Detailed counts for all PTM types are provided in Table S2, available as supplementary data at *Bioinformatics* online.

### 2.2 An example of PTM-induced structural variation

Because homologous chains are grouped only at high sequence identity, StrucPTM can highlight cases in which PTMs correspond to substantial conformational differences. For example, Src kinase structures 1Y57 and 2SRC share high sequence similarity yet exhibit low structural similarity, indicating a pronounced structural shift. The unmodified 1Y57 adopts a more open conformation, whereas phosphorylated 2SRC forms a compact, autoinhibited arrangement. These structural differences are illustrated in Fig. S2, available as supplementary data at *Bioinformatics* online.

### 2.3 Interactive website

The StrucPTM interface enables structural visualization, filtering, and comparison. Built on FastAPI + Next.js, the platform is updated weekly using the latest mmCIF, SIFTS, and UniProt releases (Burley et al. 2017, Dana et al. 2018, Consortium 2024).

Key features include global statistics, multi-parameter search, a 3Dmol.js viewer (Rego and Koes 2015) with a “Camera Mode” toggle, and TM-align-based homolog comparison (Zhang and Skolnick 2005). Furthermore, intuitive bulk download options were added; users can export search results via the “Download Table (JSON)” button or retrieve all associated structural and sequence files simultaneously using the “Download All Files (ZIP)” button.

## 3 Conclusion

StrucPTM enables robust, structure-centric investigation of PTMs through validated identification, contextual annotation, and homolog-aware comparison. This foundation supports future work in structural proteomics and PTM-aware AI models.

## Supplementary Material

btag190_Supplementary_Data

## Data Availability

The StrucPTM web interface is freely accessible at https://prix.hanyang.ac.kr/strucptm. The source code and datasets have been permanently archived on Zenodo (DOI: 10.5281/zenodo.18939125) and available on GitHub (https://github.com/HanyangBISLab/StrucPTM.git).
