# Supplementary material for: StrucPTM: a database of structurally validated protein modifications and their conformational variation
Source: Bioinformatics. 2026 Apr 22;42(5):btag190. doi: 10.1093/bioinformatics/btag190 (PMC13138842; doi:10.1093/bioinformatics/btag190)
Supplement: btag190_Supplementary_Data [file btag190_supplementary_data.zip › 260414_SupplementaryMaterial.pdf]

Supplementary Material for StrucPTM: A Database of Structurally Validated Protein Modifications and Their Conformational Variation

Seong-gwang Jeon[1], Jejoong Yoo[2], Keehyoung Joo[3], and Eunok Paek[1,*]

[1]Department of Computer Science, Hanyang University, Seoul, Korea
[2]School of Computational Sciences, Korea Institute for Advanced Study, Seoul, Korea
[3]Center for Advanced Computation, Korea Institute for Advanced Study, Seoul, Korea
[*]Corresponding author: eunokpaek@hanyang.ac.kr

Supplementary Figure S1: StrucPTM workflow

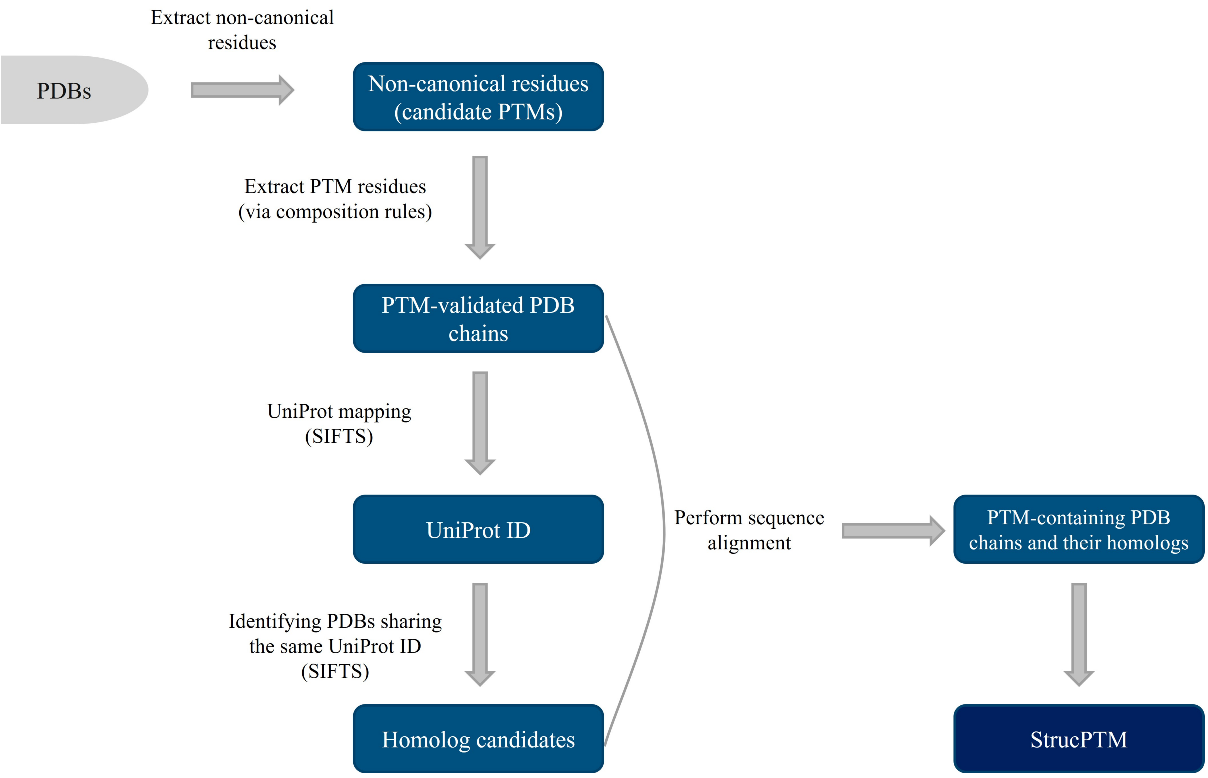

Supplementary Figure S1. Overview of the StrucPTM workflow. Non-canonical residues in PDB structures are first extracted and validated via atom-level composition rules to identify bona fide PTMs. PTM-validated PDB chains are mapped to UniProt entries (SIFTS), and homologous structures sharing the same UniProt ID are retrieved. Sequence alignment is performed to identify high-identity pairs (≥ 0.75), yielding PTM-containing structures and their homologs as the core data basis for StrucPTM.

Supplementary Figure S2: Example of PTM-induced structural change

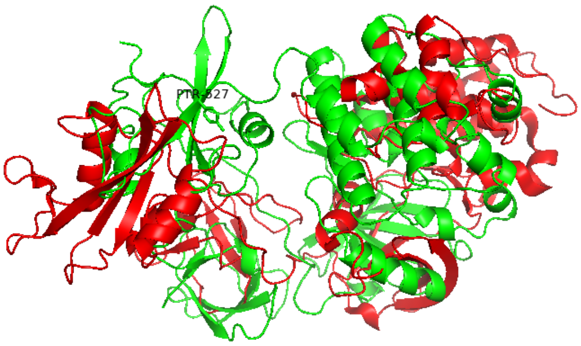

Supplementary Figure S2. Example of a phosphorylation-induced structural change. Shown is an overlay of the phosphorylated Src kinase structure (PDB: 2SRC, green) and its highly similar unmodified homolog (PDB: 1Y57, red). The phosphorylated residue Tyr-527 (PTR) is highlighted. Despite their near-identical sequence identity, the two structures adopt markedly different global conformations, illustrating how a single PTM can induce substantial structural reorganization.

Supplementary Table S1: Atom-level composition rules for PTM identification

Supplementary Table S1. Atom-level composition rules used in StrucPTM for PTM identification. StrucPTM identifies PTMs by comparing the observed atom composition of non-canonical residues with the expected chemical signatures of known PTMs, enabling systematic and annotation-independent validation.

PTM Type	Canonical Residue	Canonical Atoms	PTM Residue	Added Atoms
Acetylation	ALA	N, CA, C, O, CB	AYA	OT, CT, CM
Acetylation	LYS	N, CA, C, O, CB, CG, CD, CE, NZ	ALY	CH, CH3, OH
Acetylation	SER	N, CA, C, O, CB, OG	SAC	C2A, C1A, OAC
Formylation	MET	N, CA, C, O, CB, CG, SD, CE	FME	O1, CN
Gamma-carboxylation	GLU	N, CA, C, O, CB, CG, CD, OE1, OE2	CGU	OE21, OE22, OE11, CD2, OE12, CD1
Hydroxylation	LYS	N, CA, C, O, CB, CG, CD, CE, NZ	LYZ	OH
Hydroxylation	PRO	N, CA, C, O, CB, CG, CD	HYP	OD1
Methylation	ARG	N, CA, C, O, CB, CG, CD, NE, CZ, NH1, NH2	AGM	NE1, CE2
Methylation	ASN	N, CA, C, O, CB, CG, OD1, ND2	MEN	CE2
Methylation	CYS	N, CA, C, O, CB, SG	SMC	CS
Methylation	CYS	N, CA, C, O, CB, SG	CMT	C1
Methylation	GLN	N, CA, C, O, CB, CG, CD, OE1, NE2	MGN	CB1, CB2
Methylation	GLU	N, CA, C, O, CB, CG, CD, OE1, OE2	MEA	C1, CD2, CZ, CE2, CE1, CD1
Methylation	HIS	N, CA, C, O, CB, CG, ND1, CD2, CE1, NE2	HIC	CZ
Methylation	HIS	N, CA, C, O, CB, CG, ND1, CD2, CE1, NE2	MHS	CM
Methylation	LYS	N, CA, C, O, CB, CG, CD, CE, NZ	MLY	CH2, CH1
Methylation	LYS	N, CA, C, O, CB, CG, CD, CE, NZ	M3L	CM3, CM1, CM2
Methylation	LYS	N, CA, C, O, CB, CG, CD, CE, NZ	MLZ	CM
N6-carboxylation	LYS	N, CA, C, O, CB, CG, CD, CE, NZ	KCX	OQ2, OQ1, CX
Nitration	TYR	N, CA, C, O, CB, CG, CD1, CD2, CE1, CE2, CZ, OH	NIY	O2, NN, O1
Oxidation	CYS	N, CA, C, O, CB, SG	CSO	OD
Oxidation	MET	N, CA, C, O, CB, CG, SD, CE	OMT	OD1, OD2
Phosphorylation	SER	N, CA, C, O, CB, OG	SEP	O3P, P, O1P, O2P
Phosphorylation	THR	N, CA, C, O, CB, OG1, CG2	TPO	O3P, P, O1P, O2P
Phosphorylation	TYR	N, CA, C, O, CB, CG, CD1, CD2, CE1, CE2, CZ, OH	PTR	O3P, P, O1P, O2P
Pyro-glutamate	GLN	N, CA, C, O, CB, CG, CD, OE1, NE2	PCA	OE
S-nitrosylation	CYS	N, CA, C, O, CB, SG	SNC	OE, ND
Sulfation	TYR	N, CA, C, O, CB, CG, CD1, CD2, CE1, CE2, CZ, OH	TYS	O2, O3, S, O1

Supplementary Table S2: Comparison of PTM residues from PTM-SD and StrucPTM

Supplementary Table S2. Comparison of PTM residue counts obtained from the PTM-SD process versus StrucPTM's atom-level composition rules. StrucPTM substantially increases the number of PTM residues for most modification types.

PTM type	PTM-SD	StrucPTM
Glycosylation	20,476	127,643
Methylation	6,529	13,503
Phosphorylation	3,205	9,301
Hydroxylation	2,319	4,031
Oxidation	918	2,197
N6-carboxylysine	877	2,080
Pyrrolidone carboxylic acid	779	1,398
Gamma-carboxyglutamic acid	507	726
Acetylation	361	1,739
Formylation	293	2,681
Sulfation	254	424
Nitration	49	77
S-Nitrosylation	35	95
**Total**	**36,602**	**165,895**

Supplementary Table S3: Structural context of PTM-validated residues in StrucPTM

Supplementary Table S3. Structural context of PTM-validated residues in StrucPTM, summarizing assembly type, interfacial location, secondary structure, and base residue types for all validated PTM sites.

Statistic	Count
**Assembly type**	
Multimer	153,521
Monomer	12,374
**Residue location in multimers**	
Non-interface	119,880
Interface	33,641
**Secondary structure (DSSP)**	
C	77,376
E	31,379
T	21,714
S	20,908
H	10,638
G	2,343
B	1,076
I	461
**Base residue types**	
Asparagine	130,701
Lysine	11,234
Serine	4,791
Proline	3,980
Threonine	3,076
Methionine	2,722
Cysteine	2,626
Tyrosine	2,390
Glutamine	1,464
Histidine	1,458
Glutamic acid	891
Alanine	482
Arginine	80
