# Supplementary figures and images for: StrucPTM: a database of structurally validated protein modifications and their conformational variation
Source: Bioinformatics. 2026 Apr 22;42(5):btag190. doi: 10.1093/bioinformatics/btag190 (PMC13138842; doi:10.1093/bioinformatics/btag190)

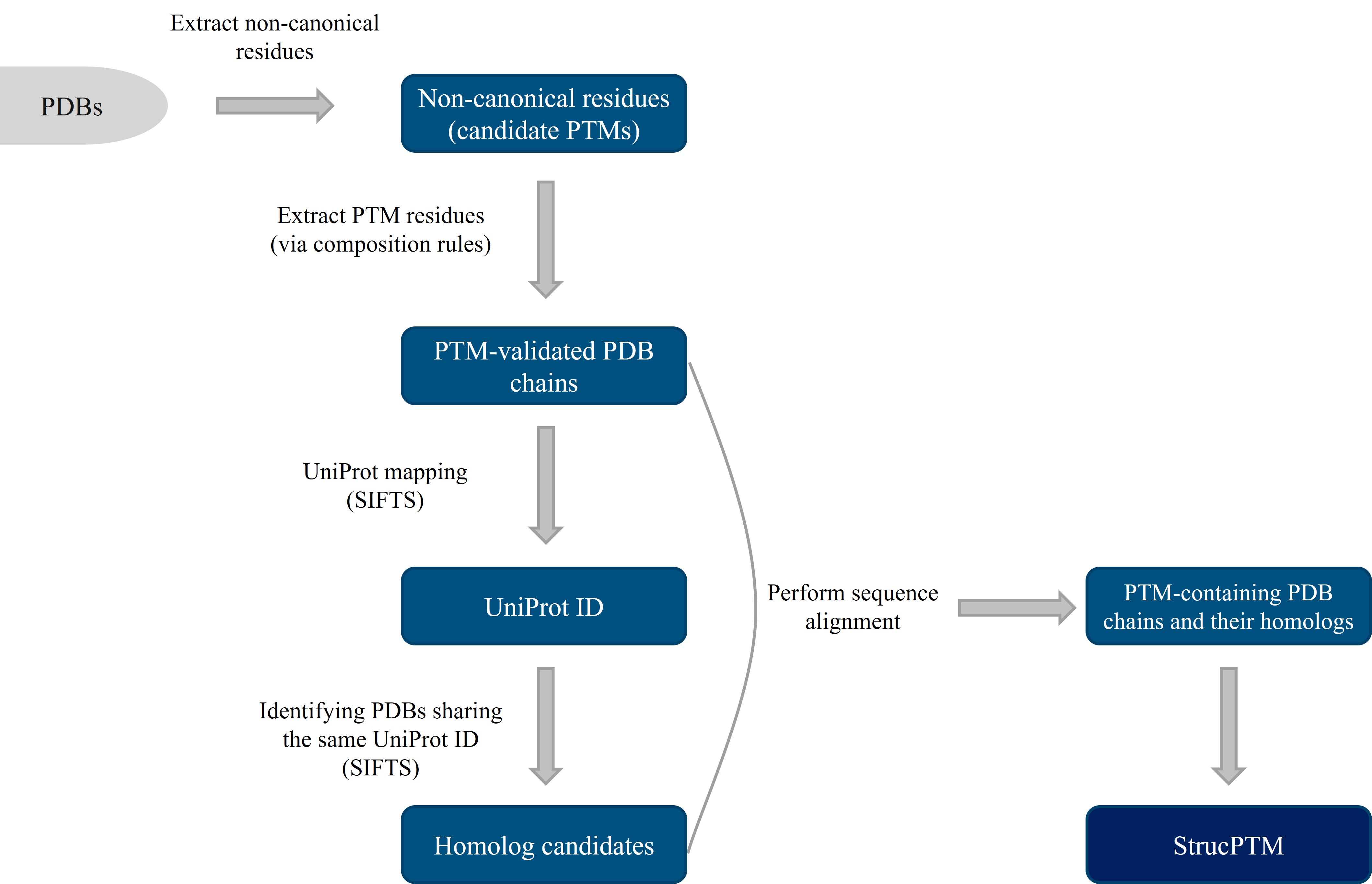

Supplement: btag190_Supplementary_Data [file btag190_supplementary_data.zip › Figure_S1.jpg]

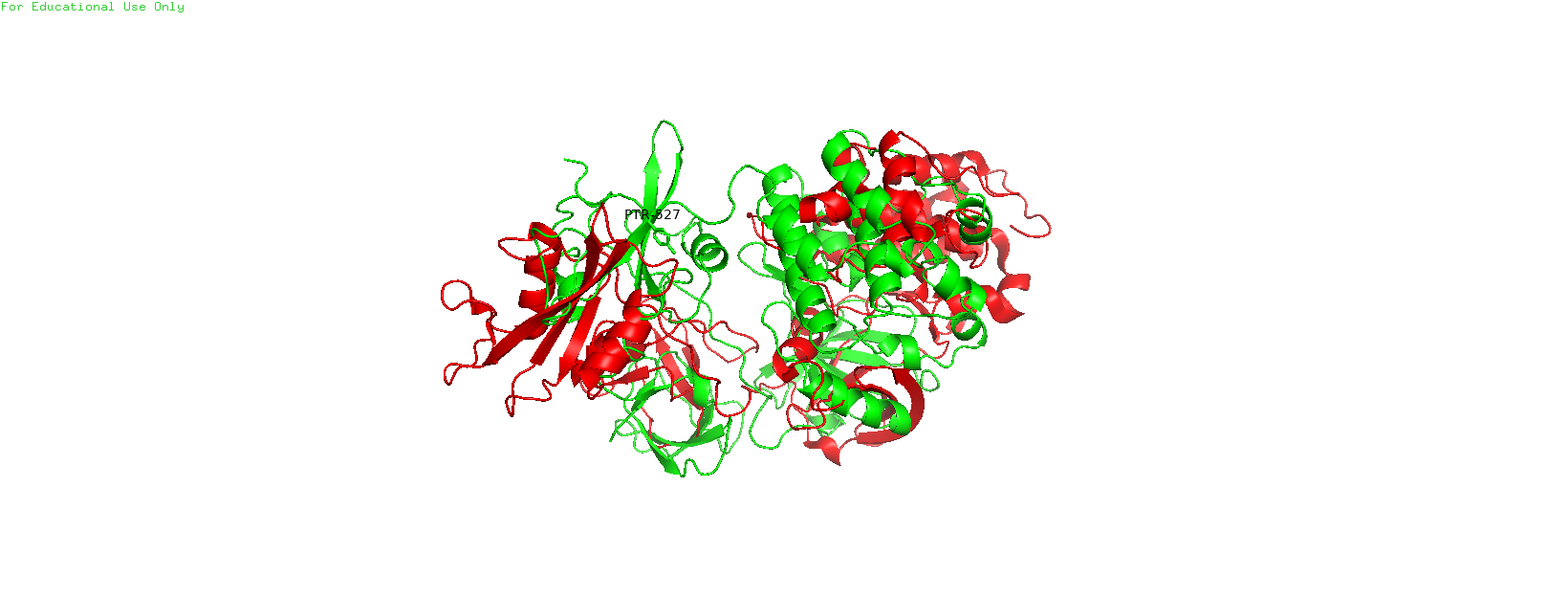

Supplement: btag190_Supplementary_Data [file btag190_supplementary_data.zip › Figure_S2.png]
